# The QardioArm Blood Pressure App for Self-Measurement in an Obese Population: Validation Study Using the European Society of Hypertension International Protocol Revision 2010

**DOI:** 10.2196/11632

**Published:** 2018-10-25

**Authors:** Victoria Mazoteras-Pardo, Ricardo Becerro-De-Bengoa-Vallejo, Marta Elena Losa-Iglesias, Daniel López-López, Patricia Palomo-López, David Rodríguez-Sanz, César Calvo-Lobo

**Affiliations:** 1 Facultad de Enfermería, Fisioterapia y Podología Universidad Complutense de Madrid Madrid Spain; 2 Faculty of Health Sciences Universidad Rey Juan Carlos Alcorcón Spain; 3 Research, Health and Podiatry Unit, Department of Health Sciences Faculty of Nursing and Podiatry Universidade da Coruña Ferrol Spain; 4 University Center of Plasencia Universidad de Extremadura Plasencia Spain; 5 Faculty of Sports Universidad Europea de Madrid Villaviciosa de Odón Spain; 6 Institute of Biomedicine, Department of Nursing and Physical Therapy Faculty of Health Sciences Universidad de León Ponferrada Spain

**Keywords:** obesity, blood pressure determination

## Abstract

**Background:**

Obesity and high blood pressure (HBP) pose high cardiovascular risks, and they are frequent causes of cardiovascular disease.

**Objective:**

The aim of this study was to validate the mobile app QardioArm for high blood pressure monitoring in obese subjects (body mass index ≥30 kg/m^2^) according to guidelines in the European Society of Hypertension-International Protocol 2 (ESH-IP2).

**Methods:**

We recruited 33 obese subjects and measured their blood pressure using QardioArm (test device) and Omron M3 Intellisense (Omron Healthcare, Kyoto, Japan; standard device). We compared systolic blood pressure (SBP), diastolic blood pressure (DBP), and heart rate (HR) according to the ESH-IP2.

**Results:**

A total of 95 of 99 differences for SBP and 91 of 99 for DBP displayed absolute differences within 10 mm Hg. A total of 98 of 99 differences for SBP and 98 of 99 for DBP exhibited absolute differences within 15 mm Hg. This result satisfied requirements for part 1 of the ESH-IP2. A total of 27 out of 33 individuals for SBP and 30 out of 33 individuals for DBP had a minimum of 2 of 3 comparisons within 5 mm Hg difference. None of the subjects had 3 differences outside 5 mm Hg for SBP and DBP, satisfying part 2 of the ESH-IP2. For HR measurements, a total of 90 of 99 differences had absolute differences within 3 beats per minute (bpm), and a total of 94 or 99 differences had absolute differences within 5 bpm. A total of 98 of 99 differences had absolute differences within 8 bpm. Therefore, the test device satisfied part 1 of ESH-IP2 criteria for HR. For part 2 of ESH-IP2, 31 of 33 individuals had a minimum of 2 of 3 comparisons within 3 bpm difference for HR. Only 1 of 33 subjects had 3 differences outside 3 bpm.

**Conclusions:**

To the best of our knowledge, this was the first study to show that an app that measures blood pressure and HR meets the requirements of the ESH-IP2 in an obese population. We believe the ESH-IP2 should publish explicit criteria for validation of blood pressure devices in specific populations.

## Introduction

Obesity and high blood pressure (HBP) pose high cardiovascular risks, and they are frequent causes of cardiovascular disease [1]. They often influence the primary causes of morbidity and mortality worldwide [2-5]. In adults, obesity is defined as body mass index (BMI) greater than 30 kg/m^2^, with overweight defined as BMI of 25 to 30 kg/m^2^, normal weight as BMI of 18.5 to 25 kg/m^2^, and underweight as BMI under 18.5 kg/m^2^[6,7]. HBP is defined as blood pressure (BP) of 140/90 mm Hg or more in adults 18 years of age or older who are not taking medication for hypertension [8-15].

The prevalence of this disease in the world is around 20% to 30%, which may increase adverse cardiovascular events in the obese population. These events are recognized by the physicians and health policy makers as significant health problems because of the several secondary impacts on the morbidity, mortality, medical, and economic cost [16,17].

Obesity and HBP are closely related, as increases in weight and BMI favor increases in BP. Conversely, weight loss reduces obesity and hypertension [9,12,18]. Causal mechanisms for obesity-associated hypertension include increased sympathetic nervous system activity, increased renal sodium retention secondary to insulin resistance or hyperinsulinemia, and obesity-mediated inflammation [19-24]. Therefore, strict BP control is necessary in obese individuals, as is weight reduction with changes in diet, lifestyle, and physical activity [24,25]. Active involvement of patients in their own treatment is a crucial factor in the successful management of hypertension. Home blood pressure monitoring (HBPM) increases patient compliance and has a great potential to improve hypertension control rates [8-12,15,24-28].

Currently, HBPM is performed with new technologies, including mobile apps, to obtain even greater benefits than can be obtained with conventional devices [29-31]. In all cases, self-measurement of BP at home requires a precise BP measurement technique and an accurate sphygmomanometer [25]. However, the primary disadvantage of automated home sphygmomanometers is their inaccuracy [32]. The majority of commercially available devices have not been evaluated independently for accuracy [13,29-31,33-37]. To evaluate the accuracy of devices in clinical practice, the Association for the Advancement of Medical Instrumentation published a validation protocol for electronic and aneroid sphygmomanometers in 1987. This was followed in 1990 by the protocol of the British Hypertension Society; both protocols were revised in 1993 [38,39]. On the basis of these experiences, the Working Group on Blood Pressure Monitoring of the European Society of Hypertension (ESH) published a simplified protocol (international protocol) to facilitate the evaluation process in 2002 that revised, unified, and simplified the previous guidelines [40]. This most recent protocol of the ESH, revised in 2010 (ESH-IP2) was more demanding than the previous protocol [32]. These protocols were meant for the general adult population, and it should not be assumed that a device that has been validated in the general population will be accurate in specific populations such as obese patients [41].

A few studies [42-44] demonstrated the accuracy of automatic BP monitors in specific populations such as obese patients, and none validated a mobile app that measures BP following the ESH protocol in this population. In 2017, QardioArm was validated in the general [45,46] and diabetic populations [46].

We hypothesized that the QardioArm mobile app for HBPM will show validated measures of BP and HR and will meet the requirements of ESH-IP2 in an obese population. Moreover, the purpose of this study was to validate the mobile app QardioArm for HBPM, according to the ESH-IP2 in obese subjects (BMI≥30 kg/m^2^) [6,7].

## Methods

### Ethics Approval

The Clinical Research Ethics Committee of Hospital Clínico San Carlos in Madrid, Spain, approved this study (number 18/135-O_P_Tesis).

This study complied with the ethical principles of the Declaration of Helsinki [47], including amendments from 2000 to 2013. Participants were informed regarding the study. All participants gave written informed consent to participate.

### The Devices

#### Omron M3 Intellisense

The Omron M3 Intellisense (Omron Healthcare, Kyoto, Japan) was the standard device used as the benchmark. The device was recently validated for the general population according to the international protocol [48]. The Omron M3 Intellisense is an automated oscillometric upper arm device for HBPM. The standard cuff of the device is for arms with circumferences of 22 to 32 cm, and a large cuff is also available for arm circumferences of 32 to 42 cm.

#### QardioArm

The QardioArm app (Qardioarm, Atten Electronic Co, Dongguan, China) was the test device. QardioArm is a fully automatic, noninvasive, and wireless BP monitor. This BP measurement system is intended to measure SBP and DBP as well as pulse rates in adults [49]. The unit uses an inflatable cuff that wraps around the upper arm. The cuff circumference is limited from 22 cm to 37 cm. To operate the device, a specific free Qardio app can be downloaded from the Apple App Store or Google Play (or getqardio website). It requires a device with Bluetooth 4.0, iOS 7.0 (or later), Android 4.4 *KitKat* (or later), and is compatible with iPhone, iPod, iPad, and Apple Watch, as well as with Android phones and tablets.

The QardioArm has an automatic screen with graphics and visuals to facilitate interpretation of data. The app can be configured to issue reminders and warnings, and the measurements and progress can be shared in real time with other users.

### Patients and Recruitment

A consecutive sampling method was used to recruit the study subjects in Ciudad Real (Spain) from family, as well as friendly and known environments to the investigator.

According to the ESH-IP2 [32], a total of 33 participants who satisfied inclusion and exclusion criteria were included. The inclusion criteria were men and women aged at least 25 years. Of the total participants, at least 10 must be men and 10 must be women, with a BMI≥30 kg/m^2^. Exclusion criteria were sustained arrhythmias, circulatory problems for which use of the cuff was contraindicated, or pregnancy.

### Study Protocol

The validation team consisted of 2 nurses with adequate experience (more than 6 years) in BP measurement. The measurement room was properly conditioned with an adequate temperature and without any factor that could influence the measurements, including noise and distractions [32,40]. Each participant reported his or her gender and date of birth, and weight, height, and BMI (calculated by the Quetelet´s equation as BMI=weight in kilograms/height in meters squared) were registered, and circumference of the arm was measured to ensure that the cuff size was adequate. Subsequently, the subject relaxed for 10 min and 9 consecutive BP measurements were performed on the same arm, with the left arm at heart level, according to the ESH-IP2 protocol [32,40]. Measurements were taken alternating the Omron M3 Intellisense and the QardioArm app, as follows:

- BPA: entry BP, with the standard device
- BPB: device detection BP with the test instrument
- BP1: with standard device
- BP2: with the test instrument
- BP3: with standard device
- BP4: with the test instrument
- BP5: with the standard device
- BP6: with the test instrument
- BP7: with the standard device

During measurement, the individual remained calm, quiet, sitting, and not moving, with the back straight, keeping the feet on the floor in a parallel position, without crossing the legs. They rested the arm on a flat surface, with the palm of the hand upwards and the elbow slightly flexed so that their arm was at the height of the heart. The interval between BP measurements was 30 to 60 seconds [32]. All measurements were made in the same room.

### Data Analysis

Statistical analyses were performed using IBM SPSS Statistics, version 19 (SPSS Inc, Chicago, Illinois) [50]. The results were expressed as mean (SD). The accuracy of a device according to the ESH-IP2 was based on comparisons between the test device (QardioArm) and the reference device (Omron M3) measurements. For each subject, the device measurements BP2, BP4, and BP6 were first compared with BP1, BP3, and BP5, respectively, and then to the measurements BP3, BP5, and BP7, respectively. Comparisons more favorable to the device were used. Differences were classified separately for both SBP and DBP according to whether the values were within 5, 10, or 15 mm Hg [32] and for the HR, according to whether the values were within 3, 5, or 8 bpm. Results were analyzed and expressed according to the ESH-IP2 requirements to determine whether the device passed or failed the validation protocol. Parts 1 and 2 of the validation process concerned the number of differences in the requested ranges for individual measurements (99 measurements) and for individual subjects (33 subjects) [32].

Bland-Altman graphs were used to represent the relationship between the difference between SBP (device to reference) and mean SBP (device and reference), DBP difference (device to reference) and mean DBP (device and reference), or HR difference (device to reference), and average HR (device and reference).

## Results

### Participants

A total of 36 subjects were screened: 14 males and 19 females. Age, weight, height, BMI, arm circumference, and their mean recruitment BP are displayed in Table 1.

### Blood Pressure Measurements

The validation results for the QardioArm BP device according to the ESH-IP2 are shown in Tables 2 and 3. The numbers of measurements differing from the standard device Omron M3 by 5, 10, and 15 mm Hg or less are displayed in Tables 2 and 3, for SBP and DBP according to the ESH-IP2 [32]. The mean differences between the standard and the tested device were 3.94 (SD 3.65) mm Hg for SBP and 3.25 (SD 3.80) mm Hg for DBP. From these analyses, a total of 71 of 99 differences for SBP presented an absolute difference within 5 mm Hg and 84 of 99 for DBP (vs at least 73 for SBP and 65 for DBP following ESH-IP2 requirements).

A total of 95 of 99 comparisons for SBP displayed an absolute difference within 10 mm Hg and 91 of 99 for DBP (vs at least 87 for SBP and 81 for DBP following ESH-IP2 requirements). A total of 98 of 99 differences for SBP exhibited an absolute difference within 15 mm Hg and 98 of 99 for DBP (vs at least 96 for SBP and 93 for DBP following ESH-IP2 requirements). These data suggest that part 1 device validation was successfully completed.

For part 2 of ESH-IP, 27 of 33 individuals had a minimum of 2 of 3 comparisons within 5 mm Hg difference for SBP and 30 of 33 subjects for DBP (vs at least 24 of 33 subjects for SBP and DBP following ESH-IP2 requirements). None of the subjects had 3 differences outside 5 mm Hg for SBP and DBP (vs a maximum of 3 subjects for SBP and DBP following ESH-IP2 requirements). Because these 2 conditions were validated, part 2 device validation was successfully completed. Thus, part 3 of the QardioArm device validation passed, as parts 1 and 2 were both validated for SBP and DBP.

**Table 1:** Sociodemographic characteristics of the participants.

Variables	Total group (n=33)		Male (n=14)		Female (n=19)	
	Mean (SD)	Range	Mean (SD)	Range	Mean (SD)	Range
Age in years	59.88 (14.97)	28-83	58.57 (15.70)	30.0-80.0	60.84 (14.76)	28.0-83.0
Weight (kg)	91.06 (11.74)	73.1-125.1	95.35 (13.43)	80.02-125.1	87.90 (9.47)	73.1-105.2
Height (cm)	167.52 (7.06)	156.0-183.0	171.00 (6.74)	160.0-183.0	164.95 (6.28)	156.0-182.0
Body mass index (kg/m^2^)	32.34 (2.33)	30.0-37.6	32.46 (2.54)	30.0-37.4	32.24 (2.23)	30.0-37.6
Arm circumference (mm)	320.70 (35.35)	275.0-370.0	308.93 (32.12)	275-370	329.37 (35.91)	280.0-369.0
Baseline systolic blood pressure	140.64 (16.38)	112.0-176.0	144.86 (16.42)	117.0-175.0	137.53 (16.08)	112.0-176.0
Baseline diastolic blood pressure	77.12 (8.51)	55.0-98.0	78.50 (9.93)	60.0-98.0	76.11 (7.41)	55.0-90.0

**Table 2:** Validation results for the QardioArm blood pressure device part 1 according to European Society of Hypertension–International Protocol 2.

Validation results QardioArm (Part 1^a^)		Criteria				
		≤5 mm Hg	≤10 mm Hg	≤15 mm Hg	Grade 1	Mean (SD)
**Pass requirements^b^**						
	Two of	73	87	96	—^c^	—
	All of	65	81	93	—	—
**Achieved^d^**						
	Systolic blood pressure	71	95	98	Pass	3.94 (3.65)
	Diastolic blood pressure	84	91	98	Pass	3.25 (3.80)

^a^Accuracy is determined by the number differences in these ranges both for individual measurements (part 1) and for individual subjects (part 2). To pass, a device must achieve all the minimum pass requirements shown.

^b^Pass requirements: as required by the international protocol.

^c^Not applicable.

^d^Achieved: as recorded by the device.

**Table 3:** Validation results for the QardioArm blood pressure device parts 2 and 3 according to European Society of Hypertension–International Protocol 2.

Validation results QardioArm (Part 2^a^)		Criteria			
		2/3 ≤5 mm Hg	0/3 ≤5 mm Hg	Grade 2	Grade 3
Pass requirements^b^		≥24	≤3	Pass	Pass
**Achieved^c^**					
	Systolic blood pressure	27	0	­—^d^	—
	Diastolic blood pressure	30	0	Pass	Pass
Part 3		—	—	—	Pass

^a^Accuracy is determined by the number differences in these ranges both for individual measurements (part 1) and for individual subjects (part 2). To pass, a device must achieve all the minimum pass requirements shown.

^b^Pass requirements: as required by the international protocol.

^c^Achieved: as recorded by the device.

^d^Not applicable.

**Table 4:** Validation results for the QardioArm heart rate device part 1 according to the European Society of Hypertension–International Protocol 2, presented as beats per minute (bmp).

Validation results QardioArm (Part 1^a^)		Criteria				
		≤3 bpm	≤5 bpm	≤8 bpm	Grade 1	Mean (SD)
**Pass requirements^b^**						
	Two of	73	87	96	—^c^	—
	All of	65	81	93	—	—
**Achieved^d^**						
	Heart rate	90	94	98	Pass	1.45 (2.04)

^a^Accuracy is determined by the number differences in these ranges both for individual measurements (part 1) and for individual subjects (part 2). To pass, a device must achieve all the minimum pass requirements shown.

^b^Pass requirements: as required by the international protocol.

^c^Not applicable.

^d^Achieved: as recorded by the device.

### Heart Rate Measurements

The validation results for the QardioArm HR device according to the ESH-IP2 are shown in Tables 4 and 5. The numbers of HR measurements differing from the standard device Omron M3 by 3, 5, and 8 bpm or less are displayed in Tables 4 and 5 for HR. The mean difference between the standard and the test device was 1.45 (SD 2.04) bpm.

From these analyses, a total of 90 of 99 differences presented absolute differences within 3 bpm, and a total of 94 of 99 comparisons displayed absolute differences within 5 bpm. A total of 98 of 99 differences exhibited absolute differences within 8 bpm. Therefore, part 1 device validation was successfully completed for HR.

For part 2 of ESH-IP, 31 of 33 individuals had a minimum of 2 of 3 comparisons within 3 bpm difference for HR. Only 1 subject had 3 differences outside 3 bpm. Since these 2 above-mentioned conditions were validated, part 2 device validation was successfully completed. Thus, part 3 of the QardioArm device validation passed, as parts 1 and 2 were both validated for HR.

**Figure 1:**
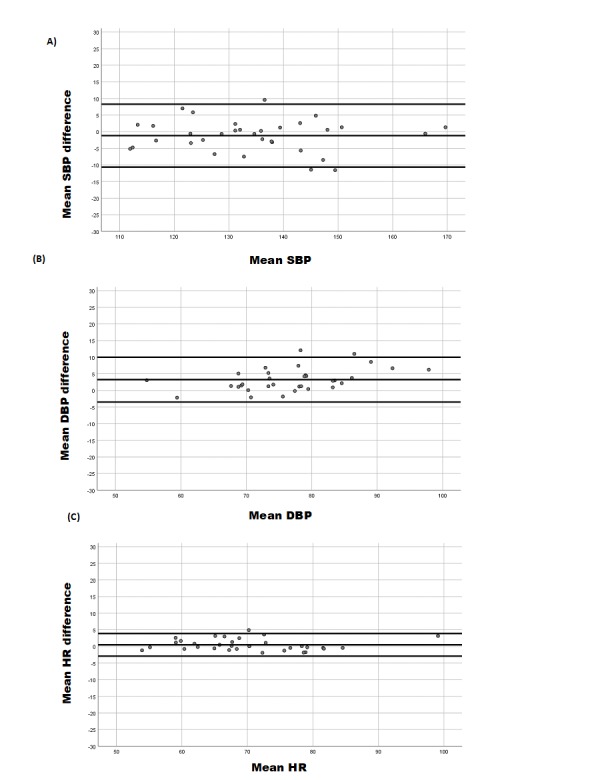
Plots of systolic blood pressure (SBP; part A), diastolic blood pressure (DBP; part B), and heart rate (HR, part C) differences between the QardioArm and the Omron M3.

In summary, the QardioArm device met validation criteria of the ESH-IP2 for SBP, DBP, and HR for subjects with BMI greater than 30 kg/m^2^. These results agreed with the Bland-Altman graphs showing differences between the measurements with the QardioArm device and the Omron M3 for SBP (Figure 1, part A), DBP (Figure 1, part B), and HR (Figure 1, part C).

**Table 5:** Validation results for the QardioArm heart rate device parts 2 and 3 according to the European Society of Hypertension–International Protocol 2, presented as beats per minute (bmp).

Validation results QardioArm (Part 2^a^)		Criteria			
		2/3 ≤5 bpm	0/3 ≤5 bpm	Grade 2	Grade 3
Pass requirements^b^		≥24	≤3	—^c^	—
**Achieved^d^**					
	Heart rate	31	1	Pass	Pass
Part 3		—	—	—	Pass

^a^Accuracy is determined by the number differences in these ranges both for individual measurements (part 1) and for individual subjects (part 2). To pass, a device must achieve all the minimum pass requirements shown.

^b^Pass requirements: as required by the international protocol.

^c^Not applicable.

^d^Achieved: as recorded by the device.

## Discussion

### Principal Findings

To the best of our knowledge, this was the first study providing information regarding the accuracy of the QardioArm app for measuring BP and HR in an obese population. This device had previously been validated in the general population by our team [45] and by others [46].

According to the ESH-IP2, we found that the QardioArm device successfully passed the validation requirements in obese population [32]. However, our results cannot be extrapolated to other specific populations, including diabetic patients, the elderly subjects, and pregnant women, as we did not address these conditions.

Although it was not the objective of this study, we observed that in obese people it was necessary to study various design aspects. Despite the efforts of the manufacturers to improve the quality of BP measuring devices, both the cuff and wrist characteristics of devices remain a point of weakness in this population [44].

In fact, although every participant was his own control, with 1 measurement recorded with QardioArm versus the reference measurement recorded by the Omron M3, BP measurement in some of our obese subjects presented some difficulties related to arms shaped more like cones than like cylinders. In some arms, the diameter at the top was larger than the diameter in the region of the brachial artery [51]. Recent findings demonstrated that despite using the appropriate cuff size, SBP appeared to be higher in subjects with bigger arms [52], suggesting that a larger limb may require greater pressure simply because there is more tissue to compress and not necessarily because there is fatty tissue. Indeed, they also observed that those with larger and more muscular arms were more likely to be misclassified as prehypertensive or hypertensive than those with smaller arms, whereas those with smaller arms may be misclassified as normal despite having elevated BP. We previously recommended that a further correction factor for arm size may be needed even when using the correct cuff size [52].

The high prevalence of obesity together with the problems introduced by large arm circumferences present risks for hypertension diagnoses [1-5,51,52]. We suggest that more validation studies should be conducted on PA devices in the obese population, as such studies are rare [42-44].

We used the ESH protocol that was published in 2002 [40] and revised in 2010 [32]. This protocol shows many advantages over previous protocols [38,39]. Nevertheless, it presents some limitations. First, the ESH-IP2 did not specify the number of validation studies that are needed to validate the instrument despite some findings reporting that a device should be validated in no fewer than 2 different centers separately [41,45,46,48]. Therefore, it is important to check the validity of BP measuring devices in specific populations as an add-on step to the validation process before widespread application in clinics or homes. Second, the specific conditions required for the recruited subjects in the study exclude children and young people, omitting data from the obese population aged between 18 and 25 years. Third, there was no mention of an explicit criterion for a validation process in specific populations in the ESH-IP, and we highly recommend that this would be taken into account in the next revision. Fourth, despite the fact that sample size calculation was conducted, the consecutive sampling bias should be considered and a simple randomization sampling process could be more adequate for future studies. Finally, despite the sphygmomanometers measure for SBP, DBP, and HR, there is no version of the International Protocol of the ESH to consider validation of HR. Therefore, validation based on the protocol criteria in BP should be added and established, in this case, the required differences based on the scale of values found after HR measurements being even more demanding than those of the ESH.

### Conclusions

To the best of our knowledge, this was the first study to show that an app that measures BP and HR meets the requirements of ESH-IP2 in an obese population. We believe the ESH-IP2 should publish explicit criteria for validation of BP devices in specific populations. Finally, we highly recommend assessment of the accuracy of this app in other specific populations such as pregnant women, elderly subjects, arrhythmic patients, and others.

## Abbreviations

BP: blood pressure
BPM: beats per minute
BMI: body mass index
DBP: diastolic blood pressure
ESH: European Society of Hypertension
ESH-IP2: European Society of Hypertension–International Protocol 2
HBP: high blood pressure
HBPM: home blood pressure monitoring
HR: heart rate
SBP: systolic blood pressure

## References

[1] Bolaji A (2014). Simulation of a real-time mobile health monitoring system model for hypertensive patient in rural Nigeria. Afr J Comp ICT.

[2] Organización Mundial de la Salud (2015). http://www.who.int/es/news-room/fact-sheets/detail/cardiovascular-diseases-(cvds).

[3] Wang H, Dwyer-Lindgren L, Lofgren KT, Rajaratnam JK, Marcus JR, Levin-Rector A, Levitz CE, Lopez AD, Murray CJ (2012). Age-specific and sex-specific mortality in 187 countries, 1970-2010: a systematic analysis for the Global Burden of Disease Study 2010. Lancet.

[4] (2015). CuidatePlus.

[5] Raghu A, Devarsetty P, Peiris D, Clifford G, Tarassenko L (2013). Engineering a mobile health tool for resource-poor settings to assess and manage cardiovascular disease risk: SMART health study. BMC Med Inform Decis Mak.

[6] American College of Cardiology/American Heart Association Task Force on Practice Guidelines, Obesity Expert Panel, 2013 (2013). Executive summary: Guidelines (2013) for the management of overweight and obesity in adults. Obesity (Silver Spring).

[7] Organización Mundial de la Salud (2017). http://www.who.int/es/news-room/fact-sheets/detail/obesity-and-overweight.

[8] Sharman JE, Howes FS, Head GA, McGrath BP, Stowasser M, Schlaich M, Glasziou P, Nelson MR (2015). Home blood pressure monitoring: Australian Expert Consensus Statement. J Hypertens.

[9] National Heart Foundation of Australia (2016). Guideline for the Diagnosis and Management of Hypertension in Adults-2016.

[10] Reboussin DM, Allen NB, Griswold ME, Guallar E, Hong Y, Lackland DT, Miller, 3rd EP, Polonsky T, Thompson-Paul AM, Vupputuri S (2017). Hypertension. Systematic Review for the 2017 ACC/AHA/AAPA/ABC/ACPM/AGS/APhA/ASH/ASPC/NMA/PCNA Guideline for the Prevention, Detection, Evaluation, and Management of High Blood Pressure in Adults: A Report of the American College of Cardiology/American Heart Association Task Force on Clinical Practice Guidelines.

[11] Daskalopoulou SS, Rabi DM, Zarnke KB, Dasgupta K, Nerenberg K, Cloutier L, Gelfer M, Lamarre-Cliche M, Milot A, Bolli P, McKay DW, Tremblay G, McLean D, Tobe SW, Ruzicka M, Burns KD, Vall&eacute;e M, Ramesh PG, Lebel M, Feldman RD, Selby P, Pipe A, Schiffrin EL, McFarlane PA, Oh P, Hegele RA, Khara M, Wilson TW, Brian PS, Burgess E, Herman RJ, Bacon SL, Rabkin SW, Gilbert RE, Campbell TS, Grover S, Honos G, Lindsay P, Hill MD, Coutts SB, Gubitz G, Campbell NR, Moe GW, Howlett JG, Boulanger J, Prebtani A, Larochelle P, Leiter LA, Jones C, Ogilvie RI, Woo V, Kaczorowski J, Trudeau L, Petrella RJ, Hiremath S, Stone JA, Drouin D, Lavoie KL, Hamet P, Fodor G, Gr&eacute;goire JC, Fournier A, Lewanczuk R, Dresser GK, Sharma M, Reid D, Benoit G, Feber J, Harris KC, Poirier L, Padwal RS (2015). The 2015 Canadian Hypertension Education Program recommendations for blood pressure measurement, diagnosis, assessment of risk, prevention, and treatment of hypertension. Can J Cardiol.

[12] Mancia G, Fagard R, Narkiewicz K, Redón J, Zanchetti A, Böhm M, Christiaens T, Cifkova R, De Backer G, Dominiczak A, Galderisi M, Grobbee DE, Jaarsma T, Kirchhof P, Kjeldsen SE, Laurent S, Manolis AJ, Nilsson PM, Ruilope LM, Schmieder RE, Sirnes PA, Sleight P, Viigimaa M, Waeber B, Zannad F, Task Force Members (2013). 2013 ESH/ESC Guidelines for the management of arterial hypertension: the Task Force for the management of arterial hypertension of the European Society of Hypertension (ESH) and of the European Society of Cardiology (ESC). J Hypertens.

[13] McLean G, Band R, Saunderson K, Hanlon P, Murray E, Little P, McManus RJ, Yardley L, Mair FS (2016). Digital interventions to promote self-management in adults with hypertension systematic review and meta-analysis. J Hypertens.

[14] James PA, Oparil S, Carter BL, Cushman WC, Dennison-Himmelfarb C, Handler J, Lackland DT, LeFevre ML, MacKenzie TD, Ogedegbe O, Smith SC, Svetkey LP, Taler SJ, Townsend RR, Wright JT, Narva AS, Ortiz E (2014). 2014 evidence-based guideline for the management of high blood pressure in adults: report from the panel members appointed to the Eighth Joint National Committee (JNC 8). J Am Med Assoc.

[15] Mancia G, De Backer G, Dominiczak A, Cifkova R, Fagard R, Germano G, Grassi G, Heagerty AM, Kjeldsen SE, Laurent S, Narkiewicz K, Ruilope L, Rynkiewicz A, Schmieder RE, Boudier HA, Zanchetti A, Vahanian A, Camm J, De Caterina R, Dean V, Dickstein K, Filippatos G, Funck-Brentano C, Hellemans I, Kristensen SD, McGregor K, Sechtem U, Silber S, Tendera M, Widimsky P, Zamorano JL, Erdine S, Kiowski W, Agabiti-Rosei E, Ambrosioni E, Lindholm LH, Viigimaa M, Adamopoulos S, Agabiti-Rosei E, Ambrosioni E, Bertomeu V, Clement D, Erdine S, Farsang C, Gaita D, Lip G, Mallion J, Manolis AJ, Nilsson PM, O'Brien E, Ponikowski P, Redon J, Ruschitzka F, Tamargo J, van Zwieten P, Waeber B, Williams B, Management of Arterial Hypertension of the European Society of Hypertension, European Society of Cardiology (2007). 2007 Guidelines for the management of arterial hypertension: the Task Force for the Management of Arterial Hypertension of the European Society of Hypertension (ESH) and of the European Society of Cardiology (ESC). J Hypertens.

[16] Hruby A, Hu FB (2015). The epidemiology of obesity: a big picture. Pharmacoeconomics.

[17] Baride JP, Kulkarni AP (2006). Text Book of Community Medicine. 3rd ed.

[18] Valenzuela AA, Solórzano-Santos F, Valenzuela AG, Durán-Arenas LG, Ponce de León-Rosales S, Oropeza MP (2016). Recomendaciones de la guía de práctica clínica de hipertensión arterial en el primer nivel de atención. Rev Med Inst Mex Seguro Soc.

[19] Lyon CJ, Law RE, Hsueh WA (2003). Minireview: adiposity, inflammation, and atherogenesis. Endocrinology.

[20] Wu H, Ghosh S, Perrard XD, Feng L, Garcia GE, Perrard JL, Sweeney JF, Peterson LE, Chan L, Smith CW, Ballantyne CM (2007). T-cell accumulation and regulated on activation, normal T cell expressed and secreted upregulation in adipose tissue in obesity. Circulation.

[21] DiBona GF (2013). Sympathetic nervous system and hypertension. Hypertension.

[22] Chen W, Leo S, Weng C, Yang X, Wu Y, Tang X (2015). Mechanisms mediating renal sympathetic nerve activation in obesity-related hypertension. Herz.

[23] Lim K, Jackson KL, Sata Y, Head GA (2017). Factors responsible for obesity-related hypertension. Curr Hypertens Rep.

[24] Landsberg L, Aronne LJ, Beilin LJ, Burke V, Igel LI, Lloyd-Jones D, Sowers J (2013). Obesity-related hypertension: pathogenesis, cardiovascular risk, and treatment: a position paper of The Obesity Society and the American Society of Hypertension. J Clin Hypertens (Greenwich).

[25] Falkner B (2017). Monitoring and management of hypertension with obesity in adolescents. Integr Blood Press Control.

[26] Parati G, Stergiou GS, Asmar R, Bilo G, de Leeuw P, Imai Y, Kario K, Lurbe E, Manolis A, Mengden T, O'Brien E, Ohkubo T, Padfield P, Palatini P, Pickering TG, Redon J, Revera M, Ruilope LM, Shennan A, Staessen JA, Tisler A, Waeber B, Zanchetti A, Mancia G (2010). European Society of Hypertension practice guidelines for home blood pressure monitoring. J Hum Hypertens.

[27] Sharman JE, Howes F, Head GA, McGrath BP, Stowasser M, Schlaich M, Glasziou P, Nelson M (2016). How to measure home blood pressure: Recommendations for healthcare professionals and patients. Aust Fam Physician.

[28] Wagner S (2017). Blood pressure self-measurement. Adv Exp Med Biol.

[29] Martínez-Pérez B, de la Torre-Díez I, López-Coronado M, Herreros-González J (2013). Mobile apps in cardiology: review. JMIR Mhealth Uhealth.

[30] Kumar N, Khunger M, Gupta A, Garg N (2015). A content analysis of smartphone-based applications for hypertension management. J Am Soc Hypertens.

[31] Omboni S, Caserini M, Coronetti C (2016). Telemedicine and m-health in hypertension management: technologies, applications and clinical evidence. High Blood Press Cardiovasc Prev.

[32] O'Brien E, Atkins N, Stergiou G, Karpettas N, Parati G, Asmar R, Imai Y, Wang J, Mengden T, Shennan A, Working Group on Blood Pressure Monitoring of the European Society of Hypertension (2010). European Society of Hypertension International Protocol revision 2010 for the validation of blood pressure measuring devices in adults. Blood Press Monit.

[33] O'Brien E, Atkins N (2007). State-of-the-market from the dableducational.org website. Blood Press Monit.

[34] Akpolat T (2010). Obesity, hypertension and home sphygmomanometer cuffs. Eur J Intern Med.

[35] Bing S, Chen K, Hou H, Zhang W, Li L, Wei J, Shu C, Wan Y (2016). Validation of the Microlife BP A200 Comfort and W2 Slim automated blood pressure monitors in a general adult population according to the European Society of Hypertension and the ANSI/AAMI/ISO 81060-2: 2013 protocols. Blood Press Monit.

[36] Dable Educational Trust (2015). http://www.dableducational.org/.

[37] Myers MG (2015). Limitations of home blood pressure monitoring in clinical practice. Can J Cardiol.

[38] Association for the Advancement of Medical Instrumentation (1993). American National Standard. Electronic or Automated Sphygmomanometers: ANSI/AAMI SP10-1993.

[39] O'Brien E, Petrie J, Littler W, de Swiet M, Padfield PL, O'Malley K, Jamieson M, Altman D, Bland M, Atkins N (1990). The British Hypertension Society protocol for the evaluation of automated and semi-automated blood pressure measuring devices with special reference to ambulatory systems. J Hypertens.

[40] O'Brien E, Pickering T, Asmar R, Myers M, Parati G, Staessen J, Mengden T, Imai Y, Waeber B, Palatini P, Gerin W, Working Group on Blood Pressure Monitoring of the European Society of Hypertension (2002). Working Group on Blood Pressure Monitoring of the European Society of Hypertension International Protocol for validation of blood pressure measuring devices in adults. Blood Press Monit.

[41] Akpolat T, Erdem E, Aydogdu T (2012). Validation of the Omron M3 Intellisense (HEM-7051-E) upper arm blood pressure monitor, for self-measurement, according to the European Society of Hypertension International Protocol revision 2010 in a stage 3-5 chronic kidney disease population. Kidney Blood Press Res.

[42] Altunkan S, Iliman N, Altunkan E (2008). Validation of the Omron M6 (HEM-7001-E) upper arm blood pressure measuring device according to the International Protocol in elderly patients. Blood Press Monit.

[43] Altunkan S, Oztaş K, Altunkan E (2006). Validation of the Omron 637IT wrist blood pressure measuring device with a position sensor according to the International Protocol in adults and obese adults. Blood Press Monit.

[44] Azaki A, Diab R, Harb A, Asmar R, Chahine MN (2017). Questionable accuracy of home blood pressure measurements in the obese population - validation of the Microlife WatchBP O3 and Omron RS6 devices according to the European Society of Hypertension-International Protocol. Vasc Health Risk Manag.

[45] Mazoteras Pardo V, Losa Iglesias ME, Lopez Chicharro J, Becerrode Bengoa Vallejo R (2017). The QardioArm app in the assessment of blood pressure and heart rate: reliability and validity study. JMIR Mhealth Uhealth.

[46] Chahine MN, Topouchian J, Zelveian P, Hakobyan Z, Melkonyan A, Azaki A, Diab R, Harb A, Asmar R (2018). Validation of BP devices QardioArm in the general population and Omron M6 Comfort in type II diabetic patients according to the European Society of Hypertension International Protocol (ESH-IP). Med Devices (Auckl).

[47] Asociación Médica Mundial (2013). https://tinyurl.com/y92zz49v.

[48] Asmar R, Khabouth J, Topouchian J, El Feghali R, Mattar J (2010). Validation of three automatic devices for self-measurement of blood pressure according to the International Protocol: The Omron M3 Intellisense (HEM-7051-E), the Omron M2 Compact (HEM 7102-E), and the Omron R3-I Plus (HEM 6022-E). Blood Press Monit.

[49] GetQardio (2016). https://www.getqardio.com/es.

[50] IBM (2010). IBM SPSS Statistics for Windows, Version 19.0.

[51] McFarlane J (2012). Blood pressure measurement in obese patients. Crit Care Nurse.

[52] Loenneke JP, Loprinzi PD, Abe T, Thiebaud RS, Allen KM, Grant Mouser J, Bemben MG (2016). Arm circumference influences blood pressure even when applying the correct cuff size: is a further correction needed?. Int J Cardiol.

